# Heterogeneous distributional responses to climate warming: evidence from rodents along a subtropical elevational gradient

**DOI:** 10.1186/s12898-017-0128-x

**Published:** 2017-04-20

**Authors:** Zhixin Wen, Yi Wu, Deyan Ge, Jilong Cheng, Yongbin Chang, Zhisong Yang, Lin Xia, Qisen Yang

**Affiliations:** 1 Key Laboratory of Zoological Systematics and Evolution, Institute of Zoology, Chinese Academy of Sciences, Beichen West Road, Beijing, 100101 China; 2 College of Life Sciences, Guangzhou University, Guangzhou, 510006 China; 3 Graduate University of Chinese Academy of Sciences, Yuquan Road, Beijing, 100049 China; 4 Institute of Rare Animals and Plants, China West Normal University, Nanchong, 637009 China

**Keywords:** Climate change, Heterogeneity, Range shift, Rodent, Species traits, Subtropical

## Abstract

**Background:**

Understanding whether species’ elevational range is shifting in response to directional changes in climate and whether there is a predictable pattern in that response is one of the major challenges in ecology. However, so far very little is known about the distributional responses of subtropical species to climate change, especially for small mammals. In this study, we examined the elevational range shifts at three range points (upper and lower range limits and abundance-weighted range centre) of rodents over a 30-year period (1986 to 2014–2015), in a subtropical forest of Southwest China. We also examined the influences of four ecological traits (body mass, habitat breadth, diet and daily activity pattern) on the upslope shifts in species’ abundance-weighted range centres.

**Results:**

Despite the warming trend between 1986 and 2015, the 11 rodent species in analysis displayed heterogeneous dynamics at each of the three range points. Species which have larger body sizes and narrower habitat breadths, show both diurnal and nocturnal activities and more specialized dietary requirements, are more likely to exhibit upslope shifts in abundance-weighted range centres.

**Conclusions:**

Species’ distributional responses can be heterogeneous even though there are directional changes in climate. Our study indicates that climate-induced alleviation of competition and lag in response may potentially drive species’ range shift, which may not conform to the expectation from climate change. Difference in traits can lead to different range dynamics. Our study also illustrates the merit of multi-faceted assessment in studying elevational range shifts.

**Electronic supplementary material:**

The online version of this article (doi:10.1186/s12898-017-0128-x) contains supplementary material, which is available to authorized users.

## Background

The past 30 years have seen an accelerating increase in the global average surface temperature, and the warming trend is still continuing [1]. One of the most striking biological impacts of ongoing climate warming is the upslope range shifts of organisms, especially when the vegetation and food resources they rely on occur successively at higher elevations [2–4]. There is a high risk of extinction for the species which are unable to keep pace with the climate change or cross the range-shift gaps [5], thus invoking a keen interest of ecologists and conservationists in elevational range shifts over recent decades.

Mountains are perhaps the best systems to investigate the interplay between climate change and species’ ranges because researchers can benefit from studying shifts in both range limits (i.e. upper and lower) of a species over relatively short spatial distances. For this reason, a substantial number of empirical studies (usually carried out at one or several mountain ranges) and meta-analyses [6, 7] have been conducted to explore species’ elevational range shifts, with the focal species including almost all the biotic groups on earth. Obviously, these studies have shown great variability in the observed responses. Notwithstanding a significant increase in temperature, species may show static distributions due to lag effect [8, 9], low mobility [10], acclimate to unfavorable climates [11] and behavioral thermoregulation instead of range shift [12]; and even counterintuitive downslope range shifts which are frequently explained by alleviation of species competition [13], water availability change [14], habitat modification [15, 16] and decreased precipitation [17]. Moreover, it is indicated that species’ range dynamics are trait-dependent. Taking small mammals as an example, some life-history and ecological traits such as longevity [18], habitat preference [19] and diet [4] have been shown to affect the responses. Considering the complexity of potential mechanisms underlying elevational range shifts, it remains a great challenge to predict the shifting direction of species. The ability to make an accurate prediction will be valuable to the evaluation of future species assemblage structures at different elevations of a mountain.

Small mammals are sensitive to environmental change [20]. To our knowledge, assessments of small mammal elevational range shifts by systematically resurveying historical sites were only seen in four elevational gradients of North America. Researchers found that small mammals showed much greater heterogeneity in range dynamics than that expected from warming [19, 21, 22]. In contrast to many other taxa, there has been little attention paid to small mammals in tropical and subtropical mountains, despite the fact that biotas here are more threatened by climate due to their generally narrower thermal niches [7, 23, 24]. Certainly, evidence from tropical and subtropical mountains is indispensable to gain an insightful understanding of the elevational range shifts of small mammals worldwide and compare the shifting directions and rates among different regions.

In this study, by revisiting historical sampling sites, we examined spatial shifts in rodent elevational ranges between historical (1986) and modern (2014–2015) times in a subtropical forest of Southwest China. Following Lenoir and Svenning [2], species’ range shift was simultaneously assessed at the upper range limit, lower range limit and range centre as their responses to climate change may differ [25, 26]. In addition, we related four species traits (body mass, habitat breadth, diet and daily activity pattern) to the upslope shifts in species’ abundance-weighted range centres. Our aims were to test (1) if species’ range shifts follow the same pattern as predicted from local climate change; and (2) if the selected traits could explain the difference in distributional responses among species.

## Methods

### Study area and climate data

The study area was an extensive elevational gradient (1550–3500 m) in the Wolong Nature Reserve (102°52′–103°24′E, 30°45′–31°25′N), Sichuan Province. Five vegetation types dominate at different elevations: evergreen broad-leaf forest (<1600 m); evergreen and deciduous mixed broad-leaf forest (1600–2100 m); coniferous and broad-leaf mixed forest (2100–2600 m); coniferous forest (2600–3100 m) and subalpine shrub and meadow (3100–3500 m). Most areas of this gradient have been fully protected since the early 1980s. Because of the extremely limited meteorological records (Dengsheng ecological station at 2800 m a.s.l., climate record available only from 1999 to 2008) in this reserve, we used climate data from the Dujiangyan meteorological station (698 m, approximately 68 km east of our study sites) to estimate the mean annual temperature (MAT) and total annual precipitation (TAP) trends between 1986 and 2015. During this period, MAT increased from 14.2 to 16.4 °C while TAP fluctuated greatly (Additional file 1: Fig. S1). Although the climate data came from a station outside our study area and were measured at a lower elevation than the sampling sites, they were the best available data in this region by far. It is also noted that MAT of Dengsheng ecological station increased by 0.6 °C between 1999 and 2008, with TAP showing little difference over time.

### Historical and modern surveys

**Fig. 1:**
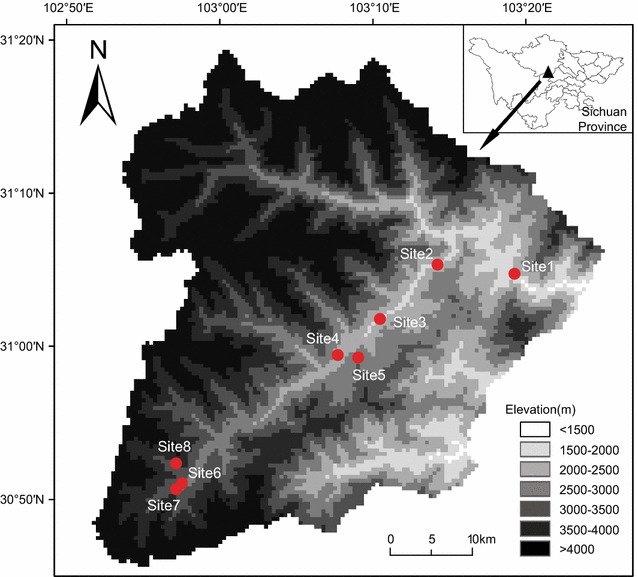
Eight sampling sites within the Wolong Nature Reserve, Sichuan Province

The historical survey was conducted at eight sites (Fig. 1) by Wu et al. [27] from March to October (each site was surveyed once every month), 1986, and the survey covered all the vegetation types along the gradient. The elevational range over which our sampling sites were distributed was 1550–3500 m. In total, 725 rodent individuals representing 18 species were captured during 11,430 trap-nights (snap traps). The skulls and voucher specimens are deposited in the Zoological Museum, China West Normal University. By examining these materials, we validated the species identification according to the taxonomic system of Wilson and Reeder [28].

In 2014–2015, we resurveyed the original sites in the same seasons (2014: July to October; 2015: March to June) and by applying the same sampling protocol and technique as in 1986. Yi Wu who led the 1986 survey contributed to the 2014–2015 resurvey by locating the sampling sites and designing the field sampling protocols. At each of the eight study sites, the difference in trapping effort (number of trap-nights) between the historical and modern surveys was less than 200 trap-nights (average difference across eight sites was 116.3 ± 19.2 trap-nights, mean ± SE). Altogether, our resurvey produced 10,500 trap-nights which resulted in the capture of 710 individuals representing 17 species. The skulls and specimen are now preserved in the Institution of Zoology, Chinese Academy of Sciences (IOZCAS). The detailed sampling information of 1986 and 2014–2015 surveys are given in Table 1.

**Table 1:** Detail information of sampling sites and sampling summary in 1986 and 2014–2015

Sampling sites	Elevation (m)	Vegetation type	Trap-nights		All individuals (species)		Eleven species individuals (species)	
			1986	2014–2015	1986	2014–2015	1986	2014–2015
1	1550	EB	2430	2300	161 (10)	193 (12)	147 (8)	186 (9)
2	1800	EDMB	1200	1200	81 (7)	179 (9)	73 (5)	176 (7)
3	1930	EDMB	1200	1100	134 (6)	73 (7)	133 (5)	72 (6)
4	2200	CBM	1220	1100	70 (7)	43 (5)	67 (4)	43 (5)
5	2500	CBM	1200	1100	65 (5)	39 (5)	65 (5)	39 (5)
6	2800	CF	1250	1100	107 (7)	100 (6)	105 (6)	99 (5)
7	3050	CF	1250	1100	43 (5)	37 (5)	40 (4)	36 (4)
8	3500	SSM	1680	1500	64 (2)	46 (2)	64 (2)	46 (2)

Vegetation type abbreviation: *EB* evergreen broad-leaf forest, *EDMB* evergreen and deciduous mixed broad-leaf forest, *CBM* coniferous and broad-leaf mixed forest, *CF* coniferous forest, *SSM* subalpine shrub and meadow

### Evaluating range shifts

We examined the shifts in upper range limit, lower range limit and abundance-weighted range centre of the 11 most common species (species with at least five captures in both periods, which formed the historical and modern datasets for comparison) between 1986 and 2014–2015. The abundance-weighted range centre of each period was calculated as:

$$\varSigma_{m,n} E_{i} \times P_{Ai}$$

where *m* and *n* were the range limits of species *A*, *E*_*i*_ was the elevation (m) of site *i* and *P*_*Ai*_ was the proportion of species *A* individuals in site *i* in its total individuals caught along the whole gradient [29].

To standardize the sampling effort, we randomly resampled (100 times without replacement) the historical and modern datasets to generate the identical number of individuals between periods at each site (e.g. 147 from the 186 individuals of 2014–2015 at site one, 72 from the 133 individuals of 1986 at site three; Table 1) [25], in the R environment (version 3.2.2). For each of the three range points, the average values derived from the 100 random resamplings of two periods were compared (modern value minus historical value) to evaluate the range shift.

To test whether an observed range shift at a given range point (range limits or centre) for a given rodent species was due to chance alone or it was a significant shift, we performed the species-level tests of significance regarding the magnitude of the observed range shift. Based on the 100 replicates of the initial datasets of two surveys, we first calculated the 100 paired differences (modern survey minus historical survey) for each of the three range points and for each of the 11 species, which produced a total of 3300 (100 × 3 × 11) elevational range shift values. For individual species, we then used three boxplots to illustrate its range shift results at different range points separately (one figure for the lower range limit, one for the lower range limit and one for the abundance-weighted range centre). Finally, each of the three boxplot was displayed against the zero reference line (i.e. no change over time) and a Student’s *t* test was conducted to determine whether the altitude of the focal point varied significantly between 1986 and 2014–2015.

### Species traits in explaining upslope shifts in range centres

We used linear regression models to examine the effect of four species traits on the upslope shifts (downslope shifts were denoted by negative values) in species’ abundance-weighted range centres, which were body mass (average mass of adults captured in 1986 and 2014–2015), habitat breadth (number of habitat types for a species; obtained from IUCN [30]), diet (categorical variable: zero for herbivores or carnivores and one for omnivores; obtained from the MammalDIET dataset by Kissling et al. [31]) and daily activity pattern [zero for obligately diurnal or nocturnal (be active only in the daytime or only at night) rodents and one for facultatively diurnal (be active mostly at night but occasionally in the daytime) rodents]. The data of species traits are provided in Additional file 1: Table S1. These traits are associated with the climate-induced range shifts for a wide range of animals, including butterflies [32], birds [33, 34] and mammals [4, 18]. Some additional traits such as litters per year, longevity and adult mobility were not tested here because data were unavailable to include these traits which were investigated in other studies [22]. Including these four traits as independent variables resulted in 15 possible models (Additional file 1: Table S2), and the best subset of models were selected by comparing their Akaike’s information criterion corrected for small sample size (AIC_C_) [35]. Because top-ranking models received nearly equivalent support (i.e., little difference in AIC_C_ values), we performed model averaging of coefficients on the models with ΔAIC_C_ ≤2 from the best model. This approach enabled us to assess the relative importance of each variable in predicting the upslope shift in range centre, according to their model-averaged standardized coefficients [36]. Model selection and model averaging were performed using the R package “MuMIn” [37].

## Results

### Species’ range shifts

**Fig. 2:**
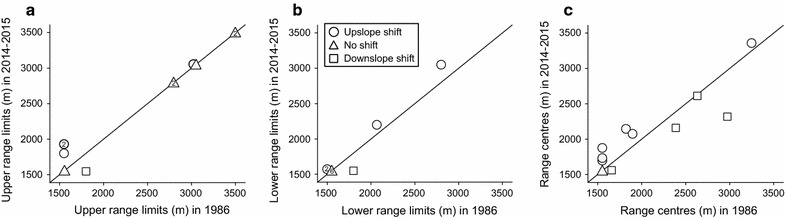
Elevational shifts of **a** upper range limits, **b** lower range limits and **c** abundance-weighted range centres of 11 rodent species between 1986 and 2014–2015. *Values* inside graphs represent the number of species showing the same range dynamics over time

Despite the general warming trend between 1986 and 2015, species’ movements at the upper range limits were heterogeneous (four upslope, six stasis, one downslope; Wilcoxon signed-rank test: n = 11, *Z* = −1.36, *P* = 0.17), and the average upslope and downslope changes were 256 ± 86 and 250 m, respectively. Similarly, there was no constant trend in the movements of lower range limits (four upslope, six stasis, one downslope; n = 11, *Z* = −0.81, *P* = 0.42; average upslope change: 101 ± 58 m, average downslope change: 250 m). Different species also showed different dynamics at the abundance-weighted range centres between periods (six upslope, one stasis, four downslope; n = 11, *Z* = −0.66, *P* = 0.51; average upslope change: 204 ± 37 m, average downslope change: 259 ± 146 m) (Fig. 2).

**Fig. 3:**
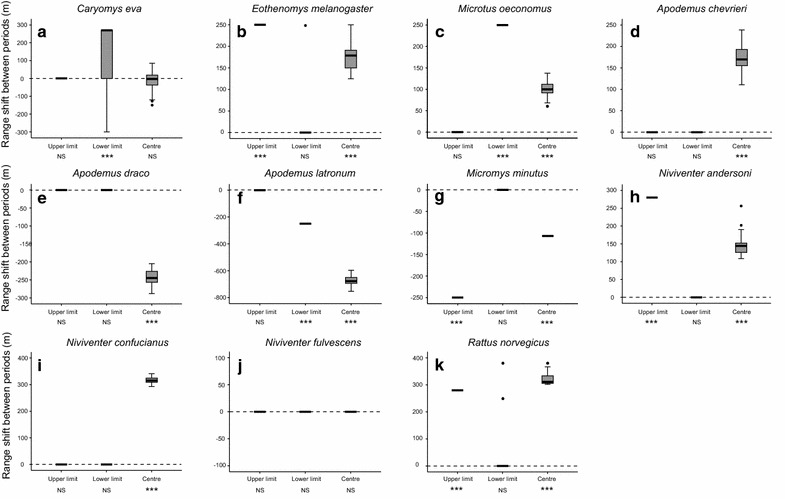
Boxplots (median and first and third quartile values are shown, outliers are denoted by *filled circles*) illustrating the elevational shifts of upper range limit, lower range limit and abundance-weighted range centre for each of 11 rodent species (**a** *Caryomys eva*, **b** *Eothenomys melanogaster*, **c** *Microtus oeconomus*, **d** *Apodemus chevrieri*, **e** *Apodemus draco*, **f** *Apodemus latronum*, **g** *Micromys minutus*, **h** *Niviventer andersoni*, **i** N*iviventer confucianus*, **j** *Niviventer fulvescens*, **k** *Rattus norvegicus*) between 1986 and 2014–2015. Elevational range shift values (n = 100) were calculated for each range point of each species as the 100 paired differences in elevation between the modern and historical surveys, based on the 100 replicates of the initial datasets of two periods. Each boxplot was displayed against the zero reference line (i.e. no shift between periods, *dotted line*) and the significance of shift was examined using a Student’s *t* test (***P < 0.001; *NS* not significant)

**Fig. 4:**
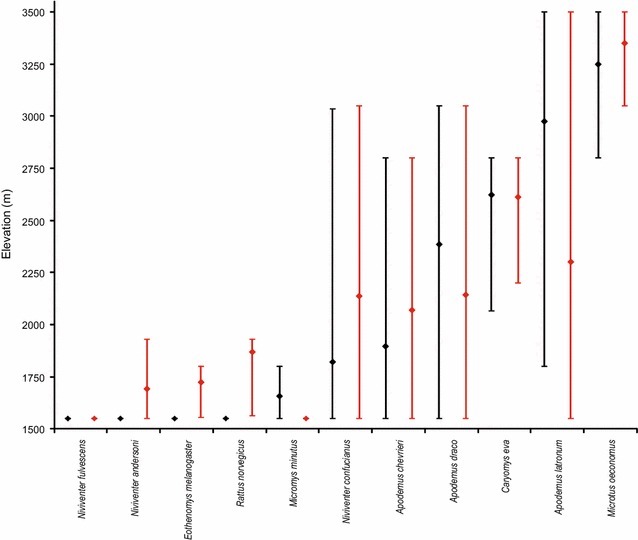
Elevational range shifts of 11 rodent species between 1986 (*black*) and 2014–2015 (*red*). The *short horizontal lines* represent the range limits and *diamonds* represent the abundance-weighted range centres. Species are arranged in ascending order of historical abundance-weighted range centre

Patterns of elevational range shift varied among species. The Student’s *t* tests of range shift for individual species showed that for the upper range limit, three (*Niviventer andersoni*, *Eothenomys melanogaster* and *Rattus norvegicus*) of six upslope shifts were found to be significant, and the only one downslope shift (*Micromys minutus*) was significant. For the lower range limit, two (*Caryomys eva* and *Microtus oeconomus*) of four upslope shifts were significant, and so was the only one downslope shift of *Apodemus latronum*. For the abundance-weighted range centre, all of the six upslope shifts were significant, while only three of four downslope shifts were significant between periods (Figs. 3, 4).

### Species traits in explaining upslope shifts in range centres

The model with the lowest AIC_C_ contained only the trait body mass. There were three models with ΔAIC_C_ ≤2 from the best model: the model contained habitat breadth alone, followed by the model contained daily activity pattern alone and the one containing diet alone (Table 2). Similarly, model averaging indicated that body mass had the highest relative importance in predicting the upslope shifts in species’ abundance-weighted range centres, followed by habitat breadth, daily activity pattern and diet (Table 2). Therefore, species which have larger body sizes and narrower habitat breadths, show both diurnal and nocturnal activities and more specialized dietary requirements, were more likely to shift their range centres towards higher elevations, although none of the traits exhibited a significant relationship with the magnitude of upslope shift in range centre.

**Table 2:** Model selection and model averaging results of models relating the upslope shifts (m) of 11 rodent species’ abundance-weighted range centres to four species traits (body mass, habitat breadth, diet and daily activity pattern), in the Wolong Nature Reserve between 1986 and 2014–2015

Model selection results					Model-averaged standardized coefficients (95% CI)			
Parameter in model	AIC_C_	ΔAIC_C_	AIC_C_ weight	*R* ^2^	Body	Habitat	Diet	Activity
Body	162.33	0	0.274	0.138	0.372	−0.314	−0.159	0.290
Habitat	162.83	0.5	0.213	0.099	−0.328 to 1.072	−1.03 to 0.402	−0.432 to 1.012	−0.903 to 0.586
Activity	163.0	0.67	0.195	0.084				
Diet	163.69	1.36	0.139	0.025				

The relationships between range shifts and different sets of trait variables were examined with generalized linear regression models, with models sorted by increasing Akaike’s information criterion (AIC_C_). Only models with ΔAIC_C_ ≤2 from the best model are shown in the table. Model-averaged standardized coefficients indicate the relative importance of four traits in predicting the upslope shifts in species’ range centres. The 95% confidence intervals are given below the standardized coefficients

## Discussion

### Heterogeneous range shifts and potential causes

There has been a growing concern about the capacity of montane biotas to track the displacement of their climatic optima, but the evidence from subtropical zones is rare [2]. Here, by comparing rodent elevational distributions across a 30-year interval, we demonstrate that despite a warming trend in a subtropical forest of China, there is heterogeneity in species’ distributional responses. Diverse patterns of range shift were revealed, including stasis, upslope and downslope range shifts. Many previous studies which reported species moving towards higher elevations in response to climate warming have also detected a portion of the investigated taxa showed no changes or downslope movements. In a global meta-analysis, Parmesan and Yohe [38] found that approximately 20% of the plant and animal species displayed downslope and southward range shifts. For plants, Lenoir et al. [39] demonstrated that in Northeast France, 53 of 171 (31%) species shifted their optimum elevations downslope between 1905–1985 and 1986–2005; and Wolf et al. [40] recently reported that merely 15% of the Californian plants’ mean elevations were higher than a century ago, and those of the rest species showed little or downslope movements. For birds, Tingley et al. [17] found that only half of the species in Sierra Nevada showed upslope shifts in upper or lower range boundary after a century of warming, possibly due to a relatively narrow elevational range over which bird eggs will hatch. As for small mammals which is our focal taxa, idiosyncratic patterns of elevational range shift have been borne out by several studies in North America [19, 21]. The disagreement between the observed range dynamics of rodents in Wolong and climatic expectations can be driven by a number of factors that shall be discussed below.

The upslope expansions of *N. andersoni*, *E. melanogaster* and *R. norvegicus*, and upslope contractions of *C. eva* and *M. oeconomus*, was probably a direct reaction to the increased temperature. As a non-native species, *R. norvegicus* may benefit from climate change in colonizing new habitats. When the temperature increases, non-native species usually tend to shift their ranges upslope to occupy the expanded potential niche space at higher elevations [40]. Temporal changes in competitive species interaction may also result in the upslope shifts. In 1986, the upper limits of *N. andersoni*, *E. melanogaster* and *R. norvegicus* were situated at 1550 m where the rodents had the highest species richness (eight species) along the gradient, implying an intense interspecific competition at this elevation with the competitors most likely to consist of ecologically similar species (e.g. three *Niviventer* species [41]; also see Additional file 1: Table S1). The competition can be provisionally alleviated by climate warming, enabling species to fill their potential distribution areas by conducting an upslope (or downslope) range shift [13]. By examining the dynamic of abundance-weighted range centre, it is possible to gain a more subtle insight into species’ distributional response. Although the change in range centre is closely linked with the change in range limits, upslope displacement of centre may manifest even though both boundaries remain unchanged, as found in *Apodemus chevrieri*. We would expect an imminent upslope range shift of the species provided the warming trend continues.

In our study, stasis was the most common dynamic (six of 11) at both range limits. By comparison, Moritz et al. [22] found that 36% of the small mammals in Yosemite National Park of California shifted their lower limits upslope. The different results between this and their research may be due to the different sampling intervals, which was a century in Moritz et al. [22]. In Wolong, the increased temperature between 1986 and 2015 may be within the tolerance ranges of some thermotolerant species. Alternatively, less vagile species may need more than 30 years to display an evident upslope shift in lower limit (i.e. lag effect [9, 25]). The stasis of the upper limits of two species, *A. latronum* and *M. oeconomus*, deserve particular attention because they were historically located at the mountaintop. The impossibility to move “higher” may have strongly affected their range dynamics and made them more vulnerable to local extinction [42]. Another interpretation of the static distributions is that species can use behavioral thermoregulation to prevent overheating, such as altering daily activity (e.g. reduce diurnal activity [21]) and hiding under vegetation shade [12]. Besides, if there are desirable habitats around at the same elevation, rodents may prefer to migrate to these nearby refugia rather than conduct an arduous shift [43], as the North American elk (*Cervus elaphus*) did in an Idaho desert [44]. Indeed, there are many cool environments in Wolong such as caves and shady valleys. These sites could facilitate the local persistence of species so that they can avoid long-distance vertical migration.

In line with many previous studies, we also observed downslope range shifts. The downslope expansion of *A. latronum* could be explained by the climate-induced alleviation of species competition [13], as long as predicable food and suitable habitats are available downwards. For *Micromys minute*, alleviated competition may be the same factor causing its downslope contraction, while changes in non-temperature factors like water availability could also underlie the movement [14]. Our result was consistent with that reported in the Great Basin, where the downslope shifts of rodents were attributed to climate-induced floristic change and land use [19].

### Range shifts related to species traits

The importance of species’ ecological traits in estimating and explaining climate-induced range shifts has been long recognized [18]. We observed a positive, albeit not statistically significant, relationship between the body mass of rodents and upslope shift in species’ abundance-weighted range centre. This finding supports the idea that compared to smaller species, larger mammal species are generally more mobile [4, 45] and characterized by better abilities to colonize a new region on the basis of higher fecundities and larger home-range sizes [46]. Intriguingly, differing from some earlier studies [18, 46, 47], habitat and dietary specialists were found showing greater upslope displacements at the range centres than generalists. We attribute our result to the strong dependence of specialists on specific prey and habitat. For example, the upslope shift in range centre of *M. oeconomus* (herbivore) could be driven by the upslope shift of its favored habitat (e.g. forest-meadow ecotone [48]) and plants. The phenomenon that habitat and dietary specialists shift upslope/northward more than generalists has also been observed in North American [33] and Central European [34] birds.

## Conclusions

Our study represents one of the first attempts to explore the climate-induced elevational range shifts of subtropical small mammals. Using a multi-faceted assessment of range shifts (upper and lower range limits, abundance-weighted range centre) for 11 rodent species, we demonstrate that the distributional responses are heterogeneous despite a general warming trend, with stasis, upslope and downslope movements all being detected. The heterogeneity is possibly due to the difference in species traits such as body mass and habitat breadth. Climate-induced alleviation of competition and lag in response may potentially drive species’ range shift, which may not conform to the expectation from climate change.

## Additional file

**Additional file 1: Table S1.** Body mass, habitat types, diet and daily activity pattern of the 11 rodent species in range shift analysis. **Table S2.** Model selection results of all 15 models relating the upslope shifts of 11 rodent species’ abundance-weighted range centres to four species traits. **Figure S1.** Changes in mean annual temperature and total annual precipitation between 1986 and 2015.

## Abbreviations

AIC_C_: Akaike’s information criterion corrected for small sample size
IOZCAS: Institution of Zoology, Chinese Academy of Sciences
MAT: mean annual temperature
TAP: total annual precipitation
