# Supplementary material for: Heterogeneous distributional responses to climate warming: evidence from rodents along a subtropical elevational gradient
Source: BMC Ecol. 2017 Apr 20;17:17. doi: 10.1186/s12898-017-0128-x (PMC5397755; doi:10.1186/s12898-017-0128-x)

**Heterogeneous distributional responses to** **climate warming: evidence from rodents along a subtropical elevational gradient**

Zhixin Wen**^1^**, Yi Wu**^2^**, Deyan Ge**^1^**, Jilong Cheng**^1^**, Yongbin Chang**^1^**, Zhisong Yang**^3^**, Lin Xia**^1^**, Qisen Yang**^1^***

**^1^** Key Laboratory of Zoological Systematics and Evolution, Institute of Zoology, Chinese Academy of Sciences, Beichen West Road, Beijing 100101, China

**^2^** College of Life Sciences, Guangzhou University, Guangzhou 510006, China

**^3^** Graduate University of Chinese Academy of Sciences, Yuquan Road, Beijing 100049, China

**^4^** Institute of Rare Animals and Plants, China West Normal University, Nanchong 637009, China

*Corresponding author: Key Laboratory of Zoological Systematics and Evolution, Institute of Zoology, Chinese Academy of Sciences, 1 Beichen West Road, Beijing, 100101, China;

yangqs@ioz.ac.cn; telephone: +86-010-64807225

**Additional file**

**Table S1.** Body mass (average mass of adults captured in 1986 and 2014–2015, mean ± SE), habitat types, diet (herbivore, carnivore and omnivore) and daily activity pattern (obligately diurnal, obligately nocturnal and facultatively diurnal) of the eleven rodent species in range shift analysis.

| Species | Body mass (g) | Habitat breadth | Diet | Daily activity pattern |
| --- | --- | --- | --- | --- |
| *Caryomys eva* | 17.0 ± 1.0 | Forest | Herbivore | Obligately nocturnal |
| *Eothenomys melanogaster* | 24.7 ± 0.7 | Forest, shrubland, cultivated land | Omnivore | Facultatively diurnal |
| *Microtus oeconomus* | 17.8 ± 0.5 | Shrubland, meadow | Herbivore | Facultatively diurnal |
| *Apodemus chevrieri* | 29.4 ± 2.2 | Forest, shrubland, cultivated land | Herbivore | Obligately nocturnal |
| *Apodemus draco* | 27.9 ± 0.7 | Forest, shrubland, cultivated land, meadow | Omnivore | Obligately nocturnal |
| *Apodemus latronum* | 27.2 ± 0.4 | Forest, shrubland, meadow | Omnivore | Obligately nocturnal |
| *Micromys minutus* | 10.5 ± 0.5 | Shrubland, cultivated land, meadow | Omnivore | Facultatively diurnal |
| *Niviventer andersoni*  *Niviventer fulvescens* | 126.1 ± 8.8  87.5 ± 2.8 | Forest  Forest, shrubland, cultivated land | Omnivore  Omnivore | Obligately nocturnal  Obligately nocturnal |
| *Niviventer confucianus* | 45.3 ± 1.1 | Forest, shrubland, cultivated land | Omnivore | Obligately nocturnal |
| *Rattus norvegicus* | 111.0 ± 9.6 | Shrubland, cultivated land | Omnivore | Facultatively diurnal |

**Table S2.** Model selection results of all possible 15 models relating the upslope shifts (m) of eleven rodent species’ abundance-weighted range centres to four species traits (body mass, habitat breadth, diet and daily activity pattern), in the Wolong Nature Reserve between 1986 and 2014–2015. The relationships between range shifts and different sets of trait variables were examined with generalized linear regression models, with models sorted by increasing Akaike’s information criterion (AIC_C_).

| Parameter in model | AIC_C_ | △AIC_C_ | AIC_C_ weight | *R*^2^ |
| --- | --- | --- | --- | --- |
| Body | 162.33 | 0 | 0.274 | 0.138 |
| Habitat | 162.83 | 0.5 | 0.213 | 0.099 |
| Activity | 163.0 | 0.67 | 0.195 | 0.084 |
| Diet | 163.69 | 1.36 | 0.139 | 0.025 |
| Body + diet | 165.95 | 3.62 | 0.045 | 0.256 |
| Body + activity | 165.97 | 3.64 | 0.044 | 0.255 |
| Habitat + activity | 167.08 | 4.75 | 0.025 | 0.176 |
| Habitat + body | 167.13 | 4.8 | 0.025 | 0.172 |
| Diet + activity | 167.89 | 5.56 | 0.017 | 0.113 |
| Habitat + diet | 168.04 | 5.71 | 0.016 | 0.101 |
| Body + diet + activity | 170.93 | 8.6 | 0.004 | 0.399 |
| Habitat + body+ activity | 172.97 | 10.64 | 0.001 | 0.277 |
| Habitat + body+ diet | 173.25 | 10.92 | 0.001 | 0.259 |
| Habitat + diet + activity | 174.37 | 12.04 | < 0.001 | 0.18 |
| Habitat + body+ diet + activity | 181.61 | 19.28 | < 0.001 | 0.417 |

**Figure S1.** Trends in (a) mean annual temperature (MAT) and (b) total annual precipitation (TAP) during 1986–2015, recorded at the Dujiangyan meteorological station (698 m a.s.l., 68 km east of the Wolong Nature Reserve).


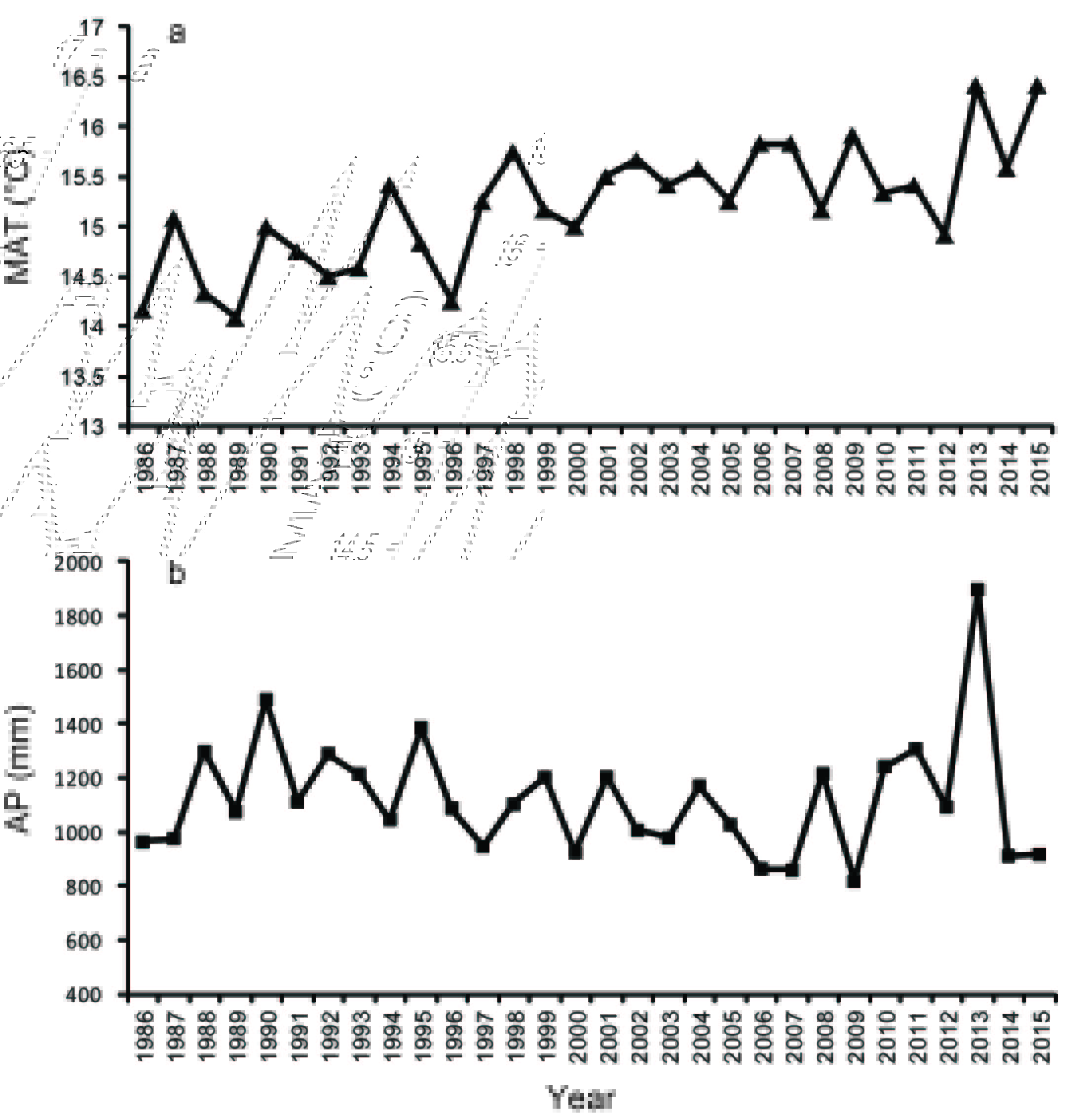

Supplement: Supplementary file 1 — Additional file 1: Table S1. Body mass, habitat types, diet and daily activity pattern of the 11 rodent species in range shift analysis. Table S2. Model selection results of all 15 models relating the upslope shifts of 11 rodent species’ abundance-weighted range centres to four species traits. Figure S1. Changes in mean annual temperature and total annual precipitation between 1986 and 2015. [file 12898_2017_128_MOESM1_ESM.docx]
